# Feasibility of using ecological momentary assessment and continuous heart rate monitoring to measure stress reactivity in natural settings

**DOI:** 10.1371/journal.pone.0264200

**Published:** 2022-03-09

**Authors:** Jessica Yang, Kiarri N. Kershaw

**Affiliations:** Department of Preventive Medicine, Northwestern University Feinberg School of Medicine, Chicago, Illinois, United States of America; University of Massachusetts Boston, UNITED STATES

## Abstract

The way people respond to stressful situations (i.e., stress reactivity) varies widely. Researchers typically measure stress reactivity in controlled studies, but this is limited because laboratory stressors cannot capture the variety, severity, or duration of stressors that individuals face in their daily lives. The present study examined the feasibility of using ecological momentary assessment (EMA) and a wireless electrocardiography (ECG) patch to develop an understanding of stress reactivity in natural settings. Thirty-five adult women completed EMA surveys about stressors they were exposed to while wearing a wireless ECG monitor for 7 consecutive days. Daily stressors were measured using seven questions adapted from the Daily Inventory of Stressful Events and a stressor interval was defined as the presence of at least one stressor during the EMA survey prompt. Participants wore the Cardea SOLO wireless ECG monitor (Cardiac Insight Inc., Bellevue, WA) to continuously track their heart rate. Participant-specific differences in 5-minute heart rate variability (HRV) between intervals when participants did and did not report stressors were calculated and displayed in a heat map. Survey response rate was satisfactory (72.0%, n = 588) and nearly all participants (33 out of 35) reported both stressor and non-stressor intervals. Each participant reported at least one stressor on approximately 35% of completed surveys while wearing the ECG patch. Mean wear time (6.6 days) and the duration of analyzable data with an ECG monitor were close to the 7-day study period. While many participants had lower HRV during stressor versus non-stressor intervals, the magnitude and direction of these differences varied widely. In summary, we found that a 7-day sampling scheme combining ecological momentary assessment (EMA) with HRV measured using continuous ECG monitoring was feasible and effective in capturing a variety of daily stressors and measuring autonomic stress reactivity.

## Introduction

While there is evidence of the contributions of stress to a variety of poor health outcomes, we know that psychosocial stressors are not internalized in the same way by everyone [1, 2]. Individuals may experience different emotional or physical changes to the same stressor, depending on how threatening they perceive the stressor to be and their available coping resources [3]. Differential stress reactivity, or how one responds to stressful situations, has been associated with deleterious health outcomes such as psychiatric disorders, cardiovascular disease, and increased hippocampal aging [3, 4].

When an individual appraises a stressor as threatening, their brain elicits a variety of emotional and physiological responses. This includes activation of the autonomic nervous system as a result of sympathovagal imbalance due to the activation of the sympathetic nervous system and the withdrawal of the parasympathetic nervous system [4, 5]. The sympathetic nervous system signals the adrenal glands to release epinephrine and cortisol, which stimulates increases in heartbeat [6], blood glucose levels [7], and respiration rate [8], as well as dilation of blood vessels in the arms and legs [9]. Similarly, parasympathetic withdrawal contributes to heart rate and blood pressure elevations [8, 10]. Although it is normal and healthy for your body to mount this type of response to a stressful situation, responses can be harmful when they do not adequately match the demands of the situation. Autonomic stress reactivity typically refers to changes in heart rate (HR) and heart rate variability (HRV) or blood pressure due to hyperactivation of the sympathetic nervous system and deactivation of the parasympathetic nervous system in response to stressors [11]. A number of recent studies [12–14] have demonstrated that greater HRV stress reactivity is an indicator of autonomic nervous system plasticity and healthy autonomic nervous system functioning. Conversely, lower HRV stress reactivity has been linked to general increased threat perception as well as depression, cardiovascular disease, and mortality [15, 16].

Researchers have typically measured HRV stress reactivity in controlled, laboratory settings in which stress responses were measured before and after the administration of an acute psychosocial stressor (e.g., Stroop task, mirror tracing, Trier Social Stress Test) [17, 18]. This methodology is limited, however, because laboratory stressors cannot capture the variety, severity, or duration of stressors that individuals face in their daily lives [4]. The few studies that have measured HRV stress reactivity in natural settings have mainly relied on 24-hour recordings of HR or HRV using a digital Holter monitor [11, 19–21]. A study by Brown et al. (2017) identified an optimal method to identify reductions in HRV due to physiological and psychological stress using a 24-hour ECG and movement data collected from an ecgMove sensor [22]. However, restricting data collection to a 24-hour period may limit the likelihood that participants will report enough moments with and without stressors to accurately measure stress reactivity in natural settings.

The objective of our pilot study was to assess the feasibility of measuring autonomic stress reactivity in a way that better captures experiences of daily stressors in natural settings. We sought to build on the existing literature by measuring stress reactivity over a 7-day period, thereby allowing for more assessments of HRV in response to stressors. In order to accomplish this, we tested a novel approach combining ecological momentary assessment (EMA) with HRV measured using continuous electrocardiography (ECG) monitoring. We examined the feasibility of employing a 7-day sampling scheme to capture a variety of daily stressors and we examined participant-specific differences in HRV during intervals when participants did and did not report being exposed to stressors.

## Methods

### Study population

We recruited 35 former participants of the Chicago Healthy Eating Environments and Resources Study (CHEERS) into our pilot study. CHEERS was a cross-sectional study of 228 non-pregnant women aged 18–44 years living in four racially, ethnically, and socioeconomically diverse neighborhoods in Chicago, Illinois [23]. Data for the original study were collected in 2016–2017, and data for this pilot study were collected from January 15, 2019 to April 9, 2019. Both the original study and the pilot study were approved by the Northwestern University Institutional Review Board, and all participants provided written informed consent to participate.

### Ecological momentary assessment

The study began by having participants complete a pre-visit questionnaire about their sleeping patterns, physical activity, health behavior decisions, and sociodemographic information. During a subsequent in-person initial visit, participants were instructed on how to download LifeData (Lifedata, LLC, Marion, IL), an EMA mobile application, on their smartphone or a study-provided smartphone. The EMA application was programmed to randomly notify the participant four times a day between the hours of 7–9am, 11am–1pm, 3–5pm, and 7–9pm for 7 consecutive days. At each notification, participants were prompted to complete a survey containing questions about their exposure to stressors. They were given 15 minutes from the time they received the first prompt to complete the survey. Participants were given up to two additional reminder prompts within the 15-minute window. Daily stressors were measured using seven items adapted from the Daily Inventory of Stressful Events, which were: had an argument, avoided an argument, had a stressor at work or school, had a stressor at home, faced discrimination, had a close friend or family member experience a stressor, or experienced any other stressor [24]. Participants responded to these items by selecting ‘Yes’ or ‘No’ to whether or not they had experienced these stressors since taking their last EMA survey. Participants were also instructed to complete a signal-contingent EMA survey each time they had a meal or snack. At the end of the EMA study period, participants completed a follow-up survey that included questions about study acceptability. Possible responses were on a 5-point Likert scale as follows: strongly disagree, disagree, neither agree nor disagree, agree, and strongly agree.

### Continuous heart rate monitoring

At the same initial study visit, participants were fitted with a Cardea SOLO wireless ECG monitor patch (Cardiac Insight Inc., Bellevue, WA) which continuously tracked their heart rate for up to 8 days. Participants were instructed not to remove nor tamper with the monitor for 7 days. In addition, participants were given an Actigraph wGT3X-BT accelerometer wristwatch and instructed to wear it on the wrist of their non-dominant hand for 7 consecutive days (ActiGraph, Pensacola, FL). Participants returned the ECG monitor and Actigraph watch during a final in-person visit approximately 7 days after the initial visit.

**Fig 1:**
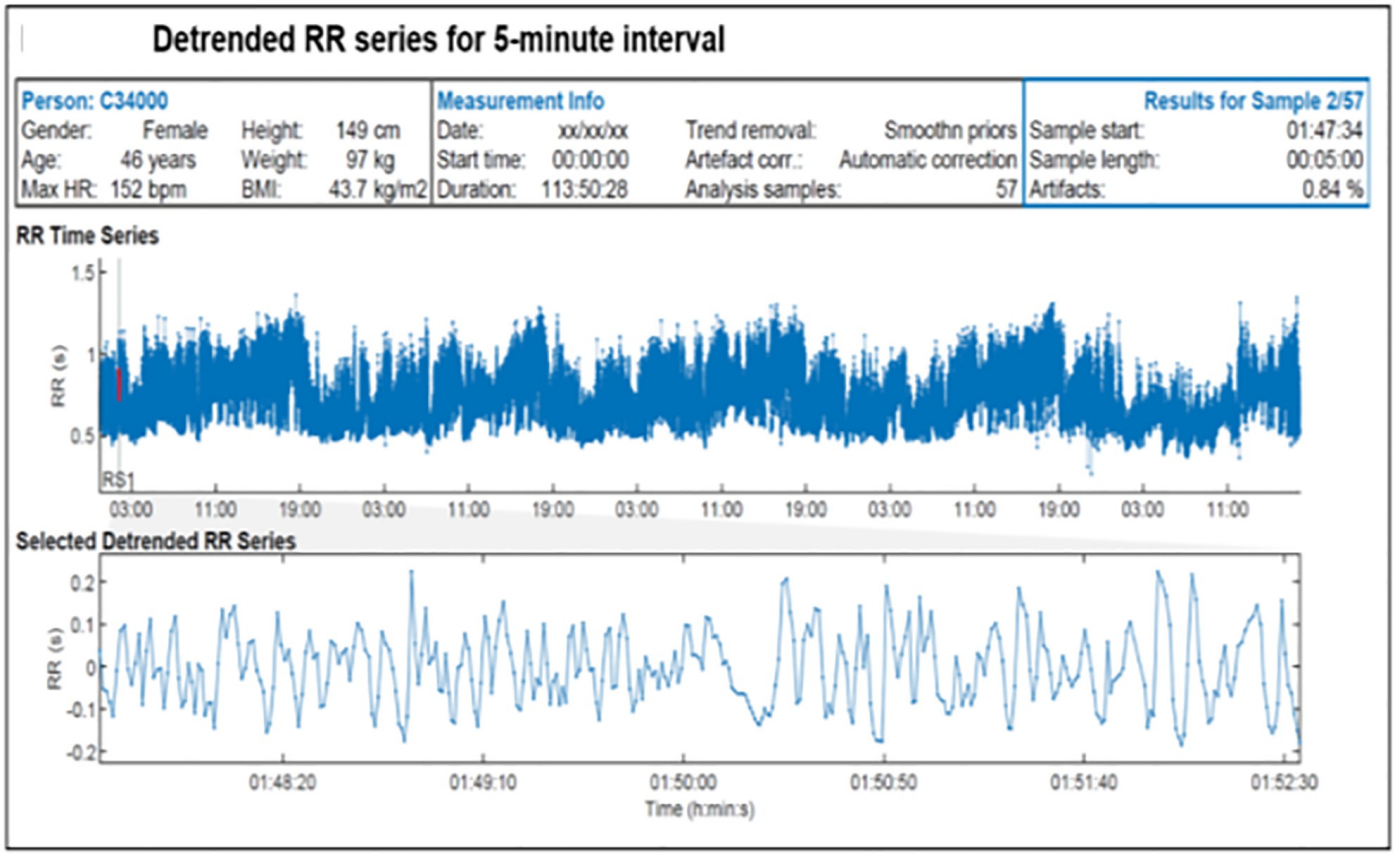
Inter-beat-interval (RR) series generated in Kubios HRV Premium software.

ECG data from the 35 returned monitors were retrieved using proprietary software (CARDEA SOLO version 2.8, ActiTrayCleaner; Cardiac Insight Inc., Bellevue, WA) (Fig 1). The data were then preprocessed using Kubios HRV Premium software (https://www.kubios.com/hrv-premium/), which uses an advanced detrending method based on smoothness priors regularization to remove slow nonstationary trends from the HRV calculations. Four measures of HRV were calculated for the 5-minute interval following the time participants started answering each EMA survey using an interval method for computing Fourier transforms [25]. The exact time when the stressor occurred is not known, so this interval was chosen since it marks the beginning of the time when participants are first recalling the stressor(s). The 5-minute interval following completion of each EMA survey was explored as an alternative timepoint.

The HRV measures calculated were root mean square of successive RR interval difference (RMSSD), standard deviation of RR intervals (STD RR), natural logarithm transformed values of absolute powers of low frequency bands (Ln LF), and natural logarithm transformed values of absolute powers of high frequency bands (Ln HF). RMSSD and STD RR are time-domain indices that quantify different aspects of the time period between successive heartbeats. Ln LF and Ln HF are frequency-domain measures that estimate the absolute power into two of four established frequency bands. All of these measures are known to be modulated by stress and are valid tools to assess autonomic activity [11, 26]. Five-minute HRV intervals with artifact levels greater than 5% were removed from the analysis in order to account for noise [27].

### Physical activity and recent eating events

Physical activity and recent meal or snack consumption may alter HRV and thus bias the measurement of autonomic stress reactivity [28, 29]. Thus, the impact of these behaviors on HRV were explored in these analyses. Physical activity was recorded by the Actigraph wristwatch and analyzed using ActiLife6 software (ActiGraph). Activity level during the 5-minute interval when HRV was measured was dichotomized as the presence or absence of a Freedson Adult (1998) Bout, which is defined as a period of time in which physical activity reaches a moderate level or greater [30]. The consumption of a recent meal or snack was dichotomized as whether or not the participant ate an hour before completing a random EMA stressor survey. This information was obtained from the timestamp of the signal-contingent surveys participants completed each time they had a meal or snack.

### Statistical analysis

First, we calculated descriptive statistics (means and proportions) for the study population. Then, we examined participant responses to questions relating to study acceptability and the distribution of reported stressors. Next, we used paired t-tests to examine the impact of non-response, sampling interval, physical activity, and recent eating, on within-participant differences in mean 5-minute HRV. Non-response was assessed by comparing mean HRV during prompts participants responded to (i.e., completed an EMA survey) vs. prompts they did not respond to (using the 5-minute interval following the first prompt participants received). Sampling interval was assessed by comparing within-participant differences in mean HRV using the 5-minute interval from when participants started answering the survey to the 5-minute interval from when they completed the survey. The impacts of physical activity and recent eating events were assessed separately by comparing mean HRV for responses that included vs. excluded these events. We used a two-tailed p value of < 0.05 to define statistical significance.

Autonomic stress reactivity has been estimated in previous studies using a mixed effects model [26, 31]. However, given the small sample size and pilot nature of our study, we instead examined differences in autonomic stress reactivity more descriptively. Specifically, we subtracted mean HRV calculated during stressor intervals (i.e., the presence of at least one stressor during the EMA survey prompt) from mean HRV calculated during non-stressor intervals for each participant and displayed results in a heat map. For each HRV measure, we broke the mean differences seen across all participants into five categories: 2+ SD below the group mean difference, 1 to <2 SD below the group mean difference, <1 SD below or above the group mean difference, 1 to <2 SD above the group mean difference, 2+ SD above the group mean difference. This approach of looking at deviations around the group mean is analogous to the mixed effects model approach used in other studies. The heat maps then display the group each participant fell into for each HRV measure. All statistical analyses were performed using or SAS version 9.4.

## Results

Mean age of our pilot study participants was 36.6 years (Table 1). The majority of participants were non-Hispanic White (n = 20; 57.1%); all other participants were either non-Hispanic Black (n = 6; 17.1%) or Hispanic/Latino (n = 9; 25.7%). Mean RMSSD, STD RR, Ln LF, and Ln HF were comparable to those measured in a sample of healthy middle-aged women in a previous study [32].

**Table 1:** Descriptive characteristics of participants.

Characteristics	Participants (n = 35)
Age (SD)	36.6 (7.2)
Race/ethnicity (n, %)	
NH White	20 (57.1)
NH Black	5 (14.3)
Hispanic/Latino	9 (25.7)
Missing	1 (2.9)
BMI	30.6 (5.7)
HRV measures (SD)	
RMSSD^a^ (ms)	31.18 (19.19)
STD RR^b^ (ms)	38.04 (16.63)
Ln LF^c^	6.39 (1.12)
Ln HF^d^	5.67 (1.47)

^a^ Root mean square of successive RR interval difference.

^b^ standard deviation of RR intervals.

^c^ natural logarithm transformed values of absolute powers of low frequency bands.

^d^ natural logarithm transformed values of absolute powers of high frequency bands.

During the period they wore the patch, participants responded to 72.0% of all EMA prompts and completed a total of 588 EMA surveys. Participants wore the ECG patch for an average of 6.6 days out of the requested 7 days. The majority (80.9%) of the 5-minute HRV intervals captured by the ECG patch during the study period were valid (i.e., artifact levels ≤ 5%). During the final in-person visit, few participants reported difficulty understanding or entering responses to the EMA surveys (Table 2). About half of the study participants found it easy to wear the heart rate monitor, while about a third did not. Less than 15% found completing the surveys to be inconvenient, and no participants reported not wanting to participate in a similar study in the future. Two participants reported experiencing a rash due to the ECG patch; those participants wore the patch for 7 days and 4 days, respectively.

**Table 2:** Participant-reported study acceptability^a^.

Statement	% Disagree or Strongly Disagree	% Agree or Strongly Agree
I had difficulty understanding the survey questions	82.9%	5.8%
I had difficulty entering my responses	94.3%	5.7%
It was easy to wear the heart rate monitor during the day	31.4%	54.3%
It was easy to wear the heart rate monitor overnight	37.1%	50.6%
Having the survey app on all the time draining my battery	80%	8.6%
Completing the survey questions was inconvenient	51.4%	14.3%
I would be willing to participate in a similar study in the future	0%	88.5%
I would recommend a similar study to a friend	8.6%	80.0%

^a^n = 35.

The median amount of time it took to complete a survey was 1 minute, 9 seconds (range from 24 seconds to 16 minutes, 22 seconds). Approximately 97% of all surveys were completed in 5 minutes or less. Of the 453 surveys with valid 5-minute HRV intervals, 165 reported at least one stressor and 288 reported no stressors. Approximately 20% of surveys had more than one reported stressor. Nearly all (33 out of 35) participants had valid stressor and non-stressor intervals, and each participant reported at least one stressor on approximately 35% of completed surveys while wearing the ECG patch. As shown in Table 3, a variety of stressors were reported. The most commonly reported stressors were avoiding an argument and experiencing a stressful event at home, which were each reported in 15.2% of all 453 surveys (41.8% of stressor intervals). The least common stressor was facing discrimination (1.3% of all surveys; 3.6% of stressor intervals).

**Table 3:** Frequencies of stressors reported.

Stressor type	Frequency (%)^a^
Had an argument	39 (8.6%)
Avoided an argument	69 (15.2%)
Stressor at work or school	48 (10.6%)
Stressor at home	69 (15.2%)
Faced discrimination	6 (1.3%)
Close friend or family member experienced a stressor	32 (7.6%)
Other stressor	64 (14.1%)

^a^Among the 453 surveys with valid HRV data.

Table 4 shows the impact of sampling interval, non-response, and recent eating and physical activity, on within-participant differences in mean valid 5-minute HRV (i.e., artifact levels ≤ 5%). The choice of sampling interval had little impact on HRV (p-values all ≥ 0.1). Mean RMSSD was slightly higher and mean Ln LF was somewhat lower in missed surveys than in completed surveys, but these differences were not statistically significant (p-values > 0.1). Mean RMSSD, STD RR, and Ln HF were all significantly higher when recent physical activity events were excluded. There were no significant differences when recent eating events were excluded (p-values > 0.1).

**Table 4:** Mean HRV by survey completion, sampling interval, physical activity, and recent eating event.

HRV measure (SD)	Completed surveys^a^ (n = 453)	Missed surveys^b^ (n = 181)	Post survey sampling interval^c^ (n = 456)	Surveys excluding activity^d^ (n = 408)	Surveys excluding recent eating event^e^ (n = 390)
RMSSD (ms)	31.18 (19.19)	32.50 (20.53)	30.91 (18.94)	32.41 (19.66)^f^	31.66 (19.18)
STD RR (ms)	38.04 (16.63)	37.59 (19.39)	37.94 (16.73)	38.75 (17.02)^f^	38.64 (16.53)
Ln LF	6.39 (1.12)	6.20 (1.30)	6.44 (1.03)	6.45 (0.99)	6.43 (1.13)
Ln HF	5.37 (1.47)	5.39 (1.57)	5.43 (1.30)	5.49 (1.34)^f^	5.40 (1.47)

^a^HRV measured in the 5-minute interval after the start of a survey.

^b^HRV measured in the 5-minute interval after the first survey prompt was received for surveys participants missed.

^c^HRV measured in the 5-minute interval following the completion of a survey.

^d^HRV measured in the 5-minute interval after the start of a survey excluding those with co-occurring Freedson Bouts.

^e^HRV measured in the 5-minute interval after the start of a survey excluding those with recent meal or snack consumption.

^f^p-value< 0.05 based on a paired t-test when compared to completed surveys.

Group mean HRV was lower in stressor intervals compared to non-stressor intervals for all measures, but participant-specific differences varied widely (Table 5). Depending on the measure, 30.3% (RMSSD) to 54.5% (ln LF) of participants had mean HRV differences > 2 SD below the group mean. A smaller percentage had mean HRV differences > 2 SD above the group mean, ranging from 12.1% for RMSSD to 18.2% for ln HF. Mean differences were in consistent categories across all 4 measures for 30.3% of participants. An additional 27.3% were in consistent categories across the two HRV time-domain measures; 24.2% were consistent across the HRV frequency-domain measures.

**Table 5:** Heat-map comparing within-participant differences in mean HRV for non-stressor intervals vs. stressor intervals^a^.

Participant^b^	RMSSD^c^	STD RR^d^	Ln LF^e^	Ln HF^f^
C10054	5.55	0.60	-0.30	0.24
C10082	2.41	2.23	0.30	0.36
C10113	-9.32	-10.91	-0.88	-0.64
C10391	-6.17	-2.73	0.06	-0.66
C10519	-9.31	-3.90	0.08	0.14
C10543	7.03	5.35	0.24	0.76
C10638	-5.47	-3.37	-0.47	-0.48
C10742	-2.03	-4.69	-0.18	-0.07
C10860	18.02	5.74	0.60	0.96
C10904	-2.76	-7.34	-0.43	0.23
C11081	-1.12	-2.17	-0.41	-0.41
C11116	2.26	4.00	0.31	0.12
C14001	-1.38	-2.89	-0.39	-0.18
C20228	8.44	6.91	0.80	0.52
C20240	-3.12	-3.27	-0.31	-0.68
C20304	4.17	1.89	-0.30	0.48
C20332	-4.04	-4.93	-0.21	0.30
C24002	-0.26	0.56	0.61	1.07
C24008	3.95	0.81	0.02	0.40
C24010	-0.07	0.82	0.10	-0.20
C24037	-21.53	-20.26	-0.75	-0.76
C30066	0.19	3.03	0.66	0.28
C30265	-10.76	-16.08	0.79	0.76
C30539	-28.37	-21.02	-0.77	-1.45
C30635	-12.83	-7.18	-0.36	-0.88
C34000	-19.63	-19.56	-0.53	-1.04
C40120	3.47	-0.53	-0.13	0.02
C40605	3.52	2.63	0.15	-0.19
C40636	-0.20	6.49	0.33	-0.21
C40661	-0.03	-2.37	-0.48	0.31
C40740	-0.17	1.03	0.10	-0.04
C40745	-1.97	-3.20	-0.88	-1.03
C40840	-3.31	-3.86	-0.42	-0.39

^a^Red cells represent values > 2 SD below the group mean HRV difference, orange cells represent 1–2 SD below the group mean HRV difference, yellow cells represent <1 SD below or above the group mean HRV difference, light green cells represent 1–2 SD above the group mean HRV difference, and dark green cells represent >2 SD above the group mean HRV difference.

^b^Participants who reported at least 1 stressor during the study period with valid HRV intervals (n = 33).

^c^Mean difference (mean HRV for non-stressor intervals—mean HRV for stressor-intervals) = 0.37 ms; SD of mean difference = 1.99 ms.

^d^Mean difference = 0.19 ms, SD of mean difference = 1.76 ms.

^e^Mean difference = 0.13, SD of mean difference = 0.12.

^f^Mean difference = 0.16, SD of mean difference = 0.15.

Because there were significant differences in mean HRV when surveys collected during physical activity bouts were excluded, we also generated a heatmap excluding those surveys (Table 6). Patterns were similar overall, with a larger percentage of participants’ differences falling > 2 SD below the group mean (33.3%–51.5%) than > 2 SD above it (15.2%–24.2%). Mean differences were in consistent categories across all 4 measures for 24.2% of participants. Differences were more consistent overall for HRV time-domain measures than HRV frequency-domain measures. An additional 36.4% of participants were in consistent categories for time-domain compared to 18.2% for frequency-domain measures.

**Table 6:** Heat-map comparing within-participant differences in mean HRV for non-stressor intervals vs. stressor intervals, excluding intervals during physical activity bouts^a^^,^^b^.

Participant^c^	RMSSD^d^	STD RR^e^	Ln LF^f^	Ln HF^g^
C10054	7.37	1.70	-0.67	0.98
C10082	3.20	2.80	0.20	0.46
C10113	-9.12	-10.30	-0.84	-0.66
C10391	-7.07	-3.37	-0.03	-0.72
C10519	-9.79	-4.76	0.05	0.14
C10543	7.03	5.35	0.24	0.76
C10638	-5.47	-3.37	-0.47	-0.48
C10742	-2.42	-5.55	-0.18	-0.09
C10860	18.02	5.74	0.60	0.96
C10904	-7.11	-9.42	-0.32	0.16
C11081	-0.35	-0.92	-0.43	-0.34
C11116	7.00	8.57	0.49	0.44
C14001	-1.38	-2.89	-0.39	-0.18
C20228	8.44	6.91	0.80	0.52
C20240	-2.52	-3.04	-0.29	-0.56
C20304	5.06	3.86	-0.17	0.63
C20332	-9.86	-7.99	-0.23	0.10
C24002	-5.61	-5.21	0.45	0.84
C24008	3.95	0.81	0.02	0.40
C24010	-0.07	0.82	0.10	-0.20
C24037	-21.53	-20.26	-0.75	-0.76
C30066	-0.14	2.43	0.53	0.14
C30265	-8.05	-10.09	0.25	0.23
C30539	-28.37	-21.02	-0.77	-1.45
C30635	-15.85	-8.40	-0.33	-1.20
C34000	-14.71	-15.35	-0.35	-0.81
C40120	3.47	-0.53	-0.13	0.02
C40605	3.52	2.63	0.15	-0.19
C40636	-1.48	4.69	0.24	-0.46
C40661	-0.03	-2.37	-0.48	0.31
C40740	-1.47	0.39	0.09	-0.10
C40745	-2.61	-3.99	-0.23	-0.51
C40840	-2.55	-1.50	-0.24	-0.34

^a^Red cells represent values > 2 SD below the group mean HRV difference, orange cells represent 1–2 SD below the group mean HRV difference, yellow cells represent <1 SD below or above the group mean HRV difference, light green cells represent 1–2 SD above the group mean HRV difference, and dark green cells represent >2 SD above the group mean HRV difference.

^b^An active interval is defined as the presence of a Freedson Bout when responding to an EMA survey.

^c^Participants who reported at least one stressor during the study period with valid HRV intervals (n = 33).

^d^Mean difference (mean HRV for non-stressor intervals—mean HRV for stressor-intervals, excluding intervals with physical activity) = 0.002 ms; SD of mean difference = 2.16 ms.

^e^Mean difference = 0.28 ms, SD of mean difference = 1.91 ms.

^f^Mean difference = 0.14, SD of mean difference = 0.11.

^g^Mean difference = 0.14, SD of mean difference = 0.15.

## Discussion

In our pilot study of adult women residing in Chicago, we found that combining EMA and continuous ECG monitoring was a feasible way to measure stress reactivity in one’s natural environment. Median wear times and analyzable times with an ECG monitor were close to the 7 days as proposed in our study protocol and EMA prompt completion was acceptable. The 7-day random EMA survey collection period was shown to adequately capture daily stressors, as nearly all participants had stressor and non-stressor intervals and a variety of different stressors were captured.

Our assessment of the impact of non-response, timing of survey interval, physical activity, and recent eating events showed that our measure of HRV was robust to most of these factors. There was no significant difference in mean HRV between surveys that were completed compared to those participants missed or those completed near an eating event. Mean HRV was also similar when using the 5-minute interval from when participants started answering the survey compared to the 5-minute interval from when they completed the survey. This is likely because most participants completed the survey in less than 5 minutes. We did see that mean HRV was significantly higher when physical activity bouts were excluded, suggesting physical activity could bias our measurement of autonomic stress reactivity. This is not unexpected given the known relationship between physical activity and HRV [33]. While our heat maps showed similar patterns overall when physical activity bouts were included vs. excluded, there were some differences. Future work in larger samples will help determine how meaningful these differences are and how best to account for physical activity in analyses (e.g., exclusion vs. statistical adjustment).

Our overall finding of lower HRV during stressor intervals compared to non-stressor intervals is consistent with previous studies that have measured changes in HRV in response to momentary distress [25, 34]. A study that assessed 219 young adults with posttraumatic stress disorder found lower ambulatory LF and HF in response to acute stressors during 24-hour EMA and ECG monitoring compared to healthy controls [26]. Another study that administered 36-hour continuous heart rate monitoring on 19 hospital workers found that daytime stress negatively correlated with the HRV measures RMSSD, HF and LF [34].

The range and variation in response that we saw among participants supports the notion that individuals react to stressors differently. Within-participant changes in HRV associated with exposure to stressors were more consistent for the time-domain measures than the frequency-domain measures. This may be because studies relating stress to HRV frequency-domain measures are more mixed. While our study and previous studies using a comparable design have demonstrated inverse associations between stressors and LF, other studies have found positive associations [11]. This heterogeneous relationship between exposure to stressors and LF may account for the less consistent within-participant differences in Ln LF and Ln HF.

This study is not without limitations. One is that we did not ask questions about severity of stressor. Another limitation is that the exact time of when participants experienced a stressor was not obtained. Accurately collecting this information is challenging since participants may not remember to note the time something stressful occurred. However, further work is needed to compare HRV at the time someone is exposed to a stressor to HRV at a time when participants are being asked to reflect on a stressor, as it is measured in our study. Another limitation is that participants did not have enough stressor events to look at differences in HRV by type of stressor or number of stressors reported. Larger studies may be better equipped to examine these differences than our pilot study.

## Conclusions

In summary, our pilot study demonstrates a promising new method of measuring autonomic stress reactivity in natural settings. Periodic random sampling of stressors via an EMA mobile app and continuous heart rate monitoring via an ECG patch over a 7-day period resulted in satisfactory participation rates and data quality. Our results capturing HRV variations among participants reflects the individualized nature of stress reactivity and further supports the feasibility of measuring stress reactivity in natural settings.

## References

[1] Hollenstein T, McNeely A, Eastabrook J, Mackey A, Flynn J. Sympathetic and parasympathetic responses to social stress across adolescence. Developmental Psychobiology. 2012;54(2):207–14. doi: 10.1002/dev.20582 21688260

[2] Piazza JR, Charles ST, Sliwinski MJ, Mogle J, Almeida DM. Affective reactivity to daily stressors and long-term risk of reporting a chronic physical health condition. Ann Behav Med. 2013;45(1):110–20. doi: 10.1007/s12160-012-9423-0 23080393 PMC3626280

[3] Carroll D, Ginty AT, Whittaker AC, Lovallo WR, de Rooij SR. The behavioural, cognitive, and neural corollaries of blunted cardiovascular and cortisol reactions to acute psychological stress. Neurosci Biobehav Rev. 2017;77:74–86. doi: 10.1016/j.neubiorev.2017.02.025 28254428 PMC6741350

[4] Epel ES, Crosswell AD, Mayer SE, Prather AA, Slavich GM, Puterman E, et al. More than a feeling: A unified view of stress measurement for population science. Front Neuroendocrinol. 2018;49:146–69. doi: 10.1016/j.yfrne.2018.03.001 29551356 PMC6345505

[5] Pal GK, Pal P, Nanda N, Amudharaj D, Adithan C. Cardiovascular dysfunctions and sympathovagal imbalance in hypertension and prehypertension: physiological perspectives. Future Cardiol. 2013;9(1):53–69. doi: 10.2217/fca.12.80 23259475

[6] Morris MC, Hellman N, Abelson JL, Rao U. Cortisol, heart rate, and blood pressure as early markers of PTSD risk: A systematic review and meta-analysis. Clin Psychol Rev. 2016;49:79–91. doi: 10.1016/j.cpr.2016.09.001 27623149 PMC5079809

[7] Geiger AM, Pitts KP, Feldkamp J, Kirschbaum C, Wolf JM. Cortisol-dependent stress effects on cell distribution in healthy individuals and individuals suffering from chronic adrenal insufficiency. Brain Behav Immun. 2015;50:241–8. doi: 10.1016/j.bbi.2015.07.010 26184081 PMC5526346

[8] Bryant RA, Creamer M, O’Donnell M, Silove D, McFarlane AC. A multisite study of initial respiration rate and heart rate as predictors of posttraumatic stress disorder. J Clin Psychiatry. 2008;69(11):1694–701. doi: 10.4088/jcp.v69n1104 19014750

[9] Carnagarin R, Kiuchi MG, Ho JK, Matthews VB, Schlaich MP. Sympathetic Nervous System Activation and Its Modulation: Role in Atrial Fibrillation. Frontiers in Neuroscience. 2019;12(1058). doi: 10.3389/fnins.2018.01058 30728760 PMC6351490

[10] Dabrowska B, Dabrowski A, Skrobowski A. Parasympathetic withdrawal precedes spontaneous blood pressure elevations in women with primary hypertension. Cardiology. 1996;87(2):119–24. doi: 10.1159/000177073 8653727

[11] Kim HG, Cheon EJ, Bai DS, Lee YH, Koo BH. Stress and Heart Rate Variability: A Meta-Analysis and Review of the Literature. Psychiatry Investig. 2018;15(3):235–45. doi: 10.30773/pi.2017.08.17 29486547 PMC5900369

[12] Fuller-Rowell TE, Williams DR, Love GD, McKinley PS, Sloan RP, Ryff CD. Race Differences in Age-Trends of Autonomic Nervous System Functioning. Journal of Aging and Health. 2013;25(5):839–62. doi: 10.1177/0898264313491427 23781017 PMC3758802

[13] Duschek S, Muckenthaler M, Werner N, del Paso GA. Relationships between features of autonomic cardiovascular control and cognitive performance. Biol Psychol. 2009;81(2):110–7. doi: 10.1016/j.biopsycho.2009.03.003 19428975

[14] Singh JP, Larson MG, Tsuji H, Evans JC, O’Donnell CJ, Levy D. Reduced Heart Rate Variability and New-Onset Hypertension. Hypertension. 1998;32(2):293–7. doi: 10.1161/01.hyp.32.2.293 9719057

[15] Thayer JF, Yamamoto SS, Brosschot JF. The relationship of autonomic imbalance, heart rate variability and cardiovascular disease risk factors. Int J Cardiol. 2010;141(2):122–31. doi: 10.1016/j.ijcard.2009.09.543 19910061

[16] Hamilton JL, Alloy LB. Atypical reactivity of heart rate variability to stress and depression across development: Systematic review of the literature and directions for future research. Clin Psychol Rev. 2016;50:67–79. doi: 10.1016/j.cpr.2016.09.003 27697746 PMC5233715

[17] dos Santos AT, Leyendecker DMD, Costa ALS, de Souza-Talarico JN. Relationship between cortisol reactivity to psychosocial stress and declarative memory decline during aging: Impact of age and sex. Geriatrics & Gerontology International. 2018;18(1):169–76. doi: 10.1111/ggi.13139 28776897

[18] Ginty AT, Phillips AC, Roseboom TJ, Carroll D, Derooij SR. Cardiovascular and cortisol reactions to acute psychological stress and cognitive ability in the Dutch Famine Birth Cohort Study. Psychophysiology. 2012;49(3):391–400. doi: 10.1111/j.1469-8986.2011.01316.x 22091868

[19] Clays E, De Bacquer D, Crasset V, Kittel F, de Smet P, Kornitzer M, et al. The perception of work stressors is related to reduced parasympathetic activity. International Archives of Occupational and Environmental Health. 2011;84(2):185–91. doi: 10.1007/s00420-010-0537-z 20437054

[20] Kimhy D, Delespaul P, Ahn H, Cai S, Shikhman M, Lieberman JA, et al. Concurrent Measurement of “Real-World” Stress and Arousal in Individuals With Psychosis: Assessing the Feasibility and Validity of a Novel Methodology. Schizophrenia Bulletin. 2009;36(6):1131–9. doi: 10.1093/schbul/sbp028 19429846 PMC2963047

[21] Kral BG, Becker LC, Blumenthal RS, Aversano T, Fleisher LA, Yook RM, et al. Exaggerated Reactivity to Mental Stress Is Associated With Exercise-Induced Myocardial Ischemia in an Asymptomatic High-Risk Population. Circulation. 1997;96(12):4246–53. doi: 10.1161/01.cir.96.12.4246 9416889

[22] Brown SBRE, Brosschot JF, Versluis A, Thayer JF, Verkuil B. New methods to optimally detect episodes of non-metabolic heart rate variability reduction as an indicator of psychological stress in everyday life. International Journal of Psychophysiology. 2018;131:30–6. doi: 10.1016/j.ijpsycho.2017.10.007 29055696

[23] Mayne SL, Jose A, Mo A, Vo L, Rachapalli S, Ali H, et al. Neighborhood Disorder and Obesity-Related Outcomes among Women in Chicago. Int J Environ Res Public Health. 2018;15(7). doi: 10.3390/ijerph15071395 29970797 PMC6069019

[24] Almeida DM, Wethington E, Kessler RC. The Daily Inventory of Stressful Events: An Interview-Based Approach for Measuring Daily Stressors. Assessment. 2002;9(1):41–55. doi: 10.1177/1073191102091006 11911234

[25] DeBoer RW, Karemaker JM, Strackee J. Comparing Spectra of a Series of Point Events Particularly for Heart Rate Variability Data. IEEE Transactions on Biomedical Engineering. 1984;BME-31(4):384–7. doi: 10.1109/TBME.1984.325351 6745974

[26] Dennis PA, Dedert EA, Van Voorhees EE, Watkins LL, Hayano J, Calhoun PS, et al. Examining the Crux of Autonomic Dysfunction in Posttraumatic Stress Disorder: Whether Chronic or Situational Distress Underlies Elevated Heart Rate and Attenuated Heart Rate Variability. Psychosom Med. 2016;78(7):805–9. doi: 10.1097/PSY.0000000000000326 27057817 PMC5003742

[27] Ribeiro GDS, Neves VR, Deresz LF, Melo RD, Dal Lago P, Karsten M. Can RR intervals editing and selection techniques interfere with the analysis of heart rate variability? Braz J Phys Ther. 2018;22(5):383–90. doi: 10.1016/j.bjpt.2018.03.008 29653903 PMC6158074

[28] James DV, Reynolds LJ, Maldonado-Martin S. Influence of the duration of a treadmill walking bout on heart rate variability at rest in physically active women. J Phys Act Health. 2010;7(1):95–101. doi: 10.1123/jpah.7.1.95 20231760

[29] Sauder KA, Johnston ER, Skulas-Ray AC, Campbell TS, West SG. Effect of meal content on heart rate variability and cardiovascular reactivity to mental stress. Psychophysiology. 2012;49(4):470–7. doi: 10.1111/j.1469-8986.2011.01335.x 22236402 PMC3403707

[30] Mâsse LC, Fuemmeler BF, Anderson CB, Matthews CE, Trost SG, Catellier DJ, et al. Accelerometer data reduction: a comparison of four reduction algorithms on select outcome variables. Med Sci Sports Exerc. 2005;37(11 Suppl):S544–54. doi: 10.1249/01.mss.0000185674.09066.8a 16294117

[31] Moretta T, Buodo G. Autonomic stress reactivity and craving in individuals with problematic Internet use. PLoS One. 2018;13(1):e0190951. doi: 10.1371/journal.pone.0190951 29338020 PMC5770068

[32] Bigger JT, Fleiss JL, Steinman RC, Rolnitzky LM, Schneider WJ, Stein PK. RR Variability in Healthy, Middle-Aged Persons Compared With Patients With Chronic Coronary Heart Disease or Recent Acute Myocardial Infarction. Circulation. 1995;91(7):1936–43. doi: 10.1161/01.cir.91.7.1936 7895350

[33] May R, McBerty V, Zaky A, Gianotti M. Vigorous physical activity predicts higher heart rate variability among younger adults. Journal of Physiological Anthropology. 2017;36(1):24. doi: 10.1186/s40101-017-0140-z 28615045 PMC5471673

[34] Uusitalo A, Mets T, Martinmäki K, Mauno S, Kinnunen U, Rusko H. Heart rate variability related to effort at work. Applied Ergonomics. 2011;42(6):830–8. doi: 10.1016/j.apergo.2011.01.005 21356531

